# Dental Management of Seven-Year-Old Child With Glanzmann Thrombasthenia: A Case Report

**DOI:** 10.7759/cureus.70243

**Published:** 2024-09-26

**Authors:** Ghaida Alduhayan, Abdulaziz Alsaif, Razan Almohareb, Mawadh Demyati

**Affiliations:** 1 Pediatric Dentistry, Prince Sultan Military Medical City, Riyadh, SAU; 2 Pediatric Dentistry, Prince Abdulrahman Advanced Dental Institute, Riyadh, SAU

**Keywords:** dental, general anesthesia, glanzmann's thrombasthenia, glycoprotein, primary dentition

## Abstract

Glanzmann thrombasthenia (GT) is an uncommon bleeding disorder that causes bleeding under the skin. Issues with platelet membrane glycoprotein IIb/IIIa are the cause of it, which makes it simpler for platelets to adhere to one another and form a thrombus. Symptoms can range from mild bruising to severe hemorrhages. Dental management of children with GT was complex, necessitating a multi-disciplinary approach in a hospital setting. The case report aimed to document a GT case involving a seven-year-old Saudi girl under general anesthesia treatment.

## Introduction

Glanzmann thrombasthenia (GT) is a rare bleeding disorder that is caused by not having enough platelet membrane glycoprotein (GP) IIb/IIIa receptors [1]. There are three types of GT based on the level of deficiency and clot retraction [2]. Patients with GT have a normal platelet count but experience prolonged bleeding and no platelet aggregation. Symptoms include mucocutaneous hemorrhage, bruising, menorrhagia, epistaxis, gingival hemorrhage, and gastrointestinal bleeding [3]. GT is a lifelong condition that can be life-threatening. GT is a genetic disorder affecting males and females equally [4]. A gene on chromosome 17 causes it. Dr. Eduard Glanzmann first documented the condition in a Swiss Alp community [5]. The incidence of GT is higher in populations with higher levels of consanguinity, which have a higher incidence of GT [6].

GT is marked by a lack of or problems with GP IIb/IIIa receptors, which are in charge of binding fibrinogen [7]. This leads to prolonged bleeding, reduced clot retraction, and suppressed platelet aggregation. Based on the level of GP IIb/IIIa, we categorize GT into three types: severe type 1, less severe type 2, and variant type 3 [8]. Accurate diagnosis using flow cytometry, monoclonal antibodies, and specific genetic tests (ITGA2B and ITGB3 genes) and treatment of GT require careful documentation of the patient’s medical history, particularly in pediatric dentistry. GT does not correlate with clinical severity and subtype, and life expectancy is within the normal range [9].

Management of dental complications in GT individuals poses many careful steps to avoid further complications [10,11]. Especially in pediatric patients, it is very important to provide safe and accurate treatment with minute precautions [12,13]. Following a dental extraction, the initial indication of GT is often extensive bleeding [14,15]. Children are typically diagnosed with GT before the age of five due to spontaneous skin bleeding [16,17]. Common symptoms include nosebleeds, straightforward bruising, gingival bleeding, gastrointestinal bleeding, and menorrhagia. Dental management in children with GT has not been reported very frequently in the literature. Therefore, the purpose of the case report was to report a case of a seven-year-old Saudi girl treated under general anesthesia.

## Case presentation

**Figure 1:**
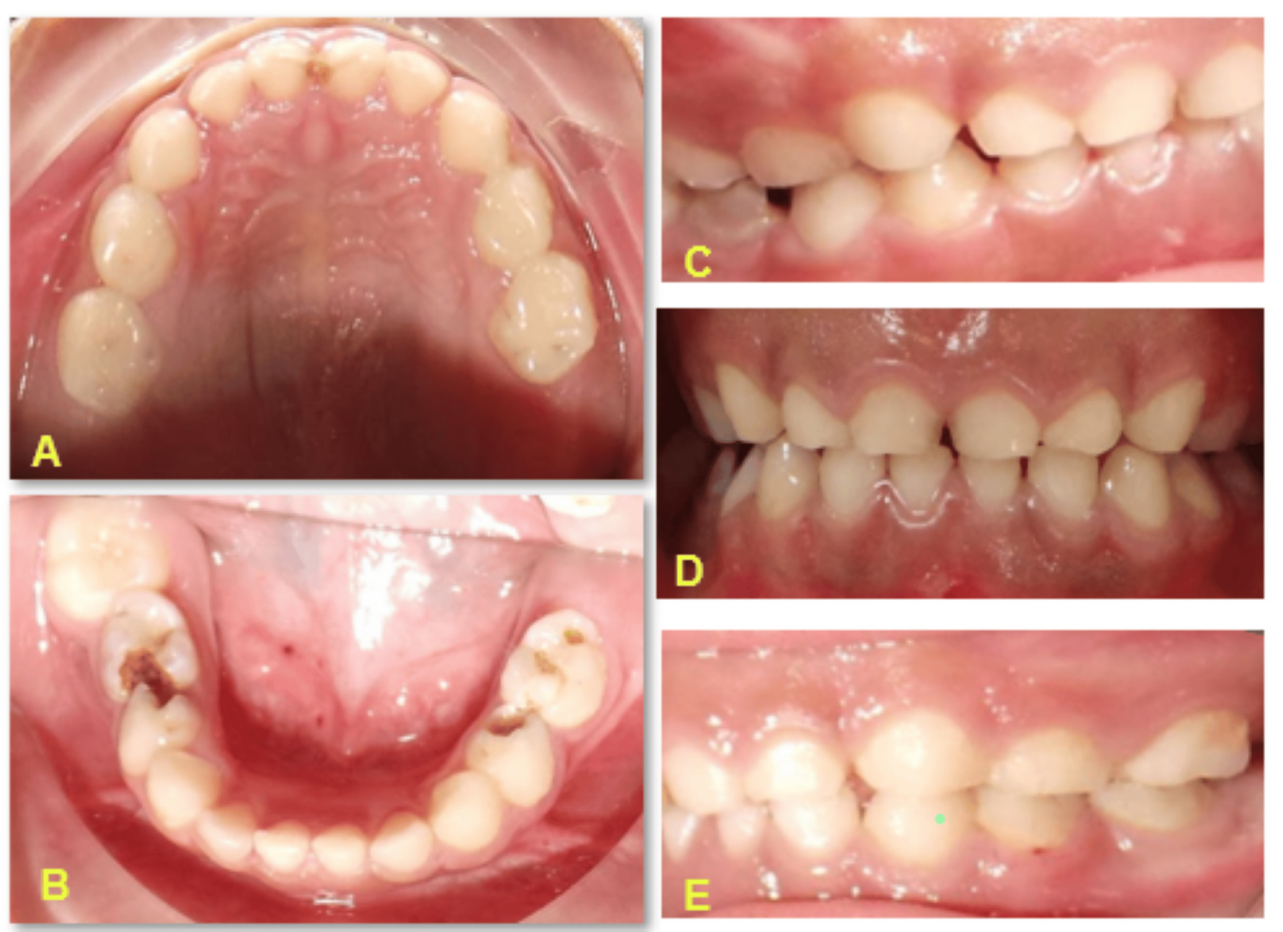
Preoperative intra-oral clinical pictures showing (a) maxillary and (b) mandibular arches, (c) left profile view, (d) frontal view, and (e) right profile views

The Department of Pediatrics referred a seven-year-old female patient with a known medical history of GT to a pediatric dentist for guidance on how to treat her bleeding gums. The patient presented with a chief complaint of severe bleeding from the upper left back tooth region since the previous night. A pediatrician referred the patient, who had a history of bleeding (>9 minutes) and low hemoglobin levels (7.5 g/dL), for dental caries management. The pediatrician advised waiting a month for recovery, leading to no dental treatment. The patient had a previous episode of bleeding and vomiting, including blood clots. There was a history of consanguineous marriage in the family. An extraoral examination revealed purpuric spots in the left leg, along with crusting and bleeding from the lips. An intraoral examination revealed primary dentition with poor oral hygiene. We observed multi-surface carious lesions in teeth 51, 61, 64, and 65 in the maxillary arch, while we reported obesity and irreversible pulpitis in teeth 75 and 85 and reversible pulpitis in teeth 74 and 84 (Figure 1).

**Figure 2:**
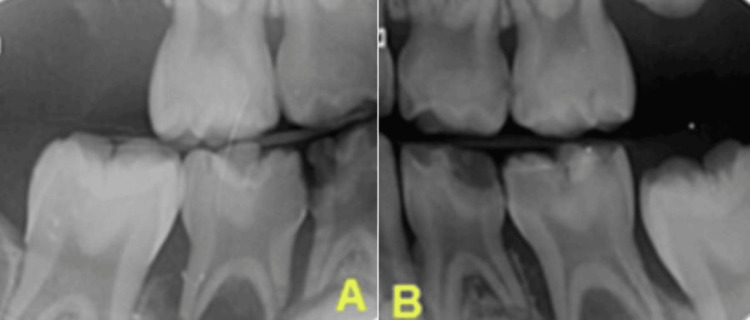
Pre-operative (a) left and (b) right molar bitewing radiographs

Both the left and right molar bitewing intra-oral radiographs confirmed the clinical finding, showing that teeth 75 and 85 had periapical pathology (Figure 2).

Gingivitis and multi-surface carious were the provisional diagnoses for teeth 51, 61, 64, and 65; chronic irreversible pulpitis for teeth 75 and 85; and reversible pulpitis for teeth 74 and 84. The child is very uncooperative; upon discussing with the parents, it was decided to perform dental treatment under general anesthesia.

Dental treatment under general anesthesia

The clearance from the pediatrician and hematologist was received prior to the dental treatment under general anesthesia. The child received 400 cc of packed red blood cells on the day of the dental treatment under general anesthesia. As a result, his hemoglobin level increased by 10 mg/dL. The planned treatment involved oral prophylaxis and the extraction of teeth 75 and 85. We recommended composite restorations for teeth 51 and 61, multi-surface carious lesions for teeth 64 and 65, stainless steel crowns for teeth 64 and 65, and pulpotomy and stainless steel crowns for teeth 74 and 84. We obtain informed consent from the parents to carry out the treatment under general anesthesia, given the child’s extreme uncooperativeness. The gingival bleeding was noticeable at the day postoperative appointment, although it was not much larger than it had been prior to treatment. We emphasized the importance of maintaining good oral hygiene, using a soft-bristled toothbrush with fluoridated toothpaste, and brushing using the Fones technique. The child was scheduled for the dental procedure on the fourth day. 

Instructions

Diet counseling was done. The patient advocated for stringent oral hygiene instructions. We advised the patient to use a soft-bristled toothbrush and fluoridated toothpaste and gargle after every meal. We also advised the parent to use a soft splint with tranexamic acid paste whenever bleeding occurred. Avoid hot, hard, and spicy food, and only a cold and soft diet for the next five days was suggested and informed to the parents.

Follow-up and outcome

**Figure 3:**
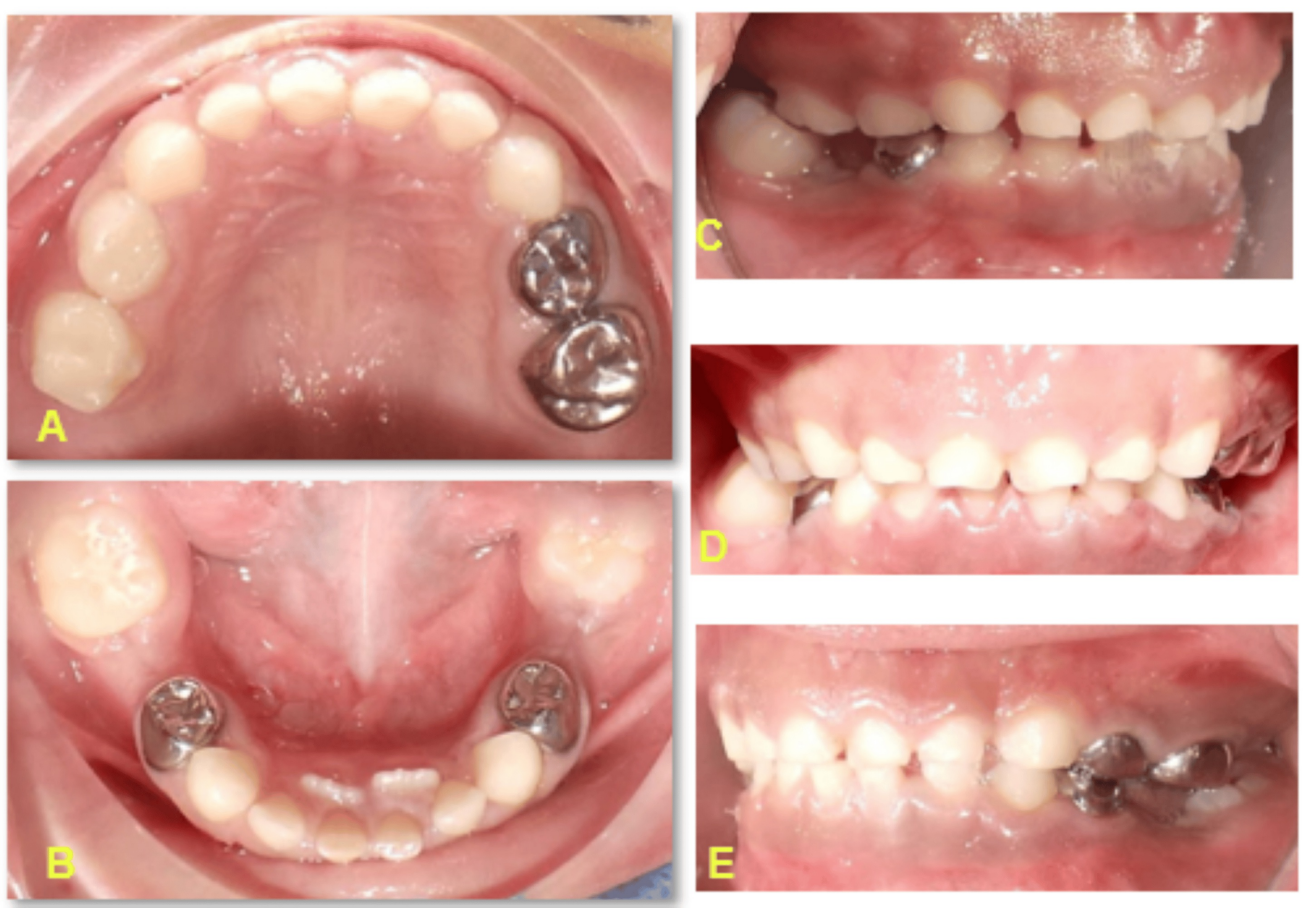
Post-operative intra-oral clinical pictures showing (a) maxillary and (b) mandibular arches, (c) left profile view, (d) frontal view, and (e) right profile views

Postoperatively, a two-week follow-up revealed no active bleeding from the gums, but there was satisfactory healing at the extraction site. We reviewed the patient every two months for six months. The subsequent follow-up visits involved placing sealants on tooth 46. Six months later, lingually erupted teeth 31 and 41 were observed next to teeth 71 and 81 (Figure 3).

**Figure 4:**
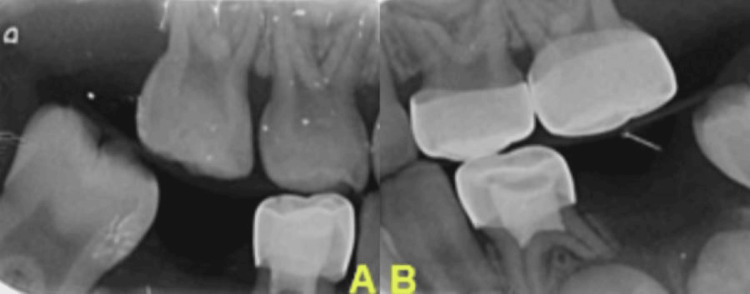
Post-operative (a) left and (b) right molar bitewing radiographs after 6 months

The child remains vigilant for natural exfoliation of mandibular primary incisors. At the six-month follow-up, there were no active carious lesions, no active bleeding, and no history of hospitalization. The six-month follow-up left and right bitewing radiographs showed confirmed clinical findings (Figure 4).

## Discussion

GT is a rare genetic disorder that affects blood coagulation due to platelet dysfunction. In areas where there is intermarriage, it is more prevalent, according to pediatrician Glanzmann’s 1918 report [1]. The incidence of GT is about one in 1,000,000 people [18,19]. Symptoms include bleeding from the gums, nose, and skin, as well as spontaneous loss of teeth [19]. Blood transfusion may be necessary to prevent excessive bleeding [3,4]. Dental treatment for GT patients should focus on preventing hemorrhages and maintaining oral hygiene to prevent gingivitis and dental caries [12,15]. Dentists should modify their examination and treatment plans to minimize bleeding during procedures. Regular checkups and oral hygiene practices are essential for GT patients [11,13,16].

GT is an uncommon hemorrhagic condition resulting from abnormalities in the GP IIb/IIIa complex located on the platelet membrane [5]. Platelet transfusion is the prevailing treatment for this condition. Earlier reports showed that repeated transfusions can cause autoimmunization, which renders transfusions ineffective [6]. Preventing platelet immunization is crucial for managing bleeding and preventing neonatal thrombocytopenia. However, rFVII is not universally effective, and platelet transfusions are essential in high-risk situations. Local interventions like cautery and fibrin glue are affordable and efficient. There is no consistent medication for GT [20]. A multidisciplinary team approach, including pediatricians, hematologists, and pediatric dentists, is necessary for effective management. Regular dental visits and maintaining good oral hygiene can prevent multiple transfusions. Raising awareness among healthcare providers about the importance of regular dental visits is essential to prevent potentially life-threatening situations [11,13,14,16,17,20].

Children with special needs may receive treatment using various psychological methods that are tailored to their cooperative lives. Patients with special needs could receive safe dental treatment under general anesthesia [21,22]. Bahadure et al. [16] argue that the pediatric dentist must be aware of bleeding disorders when evaluating continuous and prolonged bleeding from minor injuries in playful preschool children. According to a report by Jasmin et al. [11], people who have GT tend to bleed. However, the authors stated that children with the disease can have extractions done successfully without the need for additional care. It has been reported that a large number of blood products are used in such procedures, including the use of recombinant activated factor VII [19]. Similarly, in the present case, the child was treated without additional care. Ghosh et al. [17] said that they thought the case report showed a way to treat total exodontia in people with severe GT who have chronic spontaneous gingival bleeding, which causes their hemoglobin levels to stay low. The postoperative raised and maintained levels of hemoglobin on follow-up substantiate our treatment protocol [15]. No literature has yet reported such a radical treatment. However, in the present instance, we only extracted two mandibular primary molars and preferred to adopt a conservative approach instead of pursuing an aggressive strategy of extracting all teeth. The literature recommends that adopting a conservative approach for special needs children can enhance their quality of life through consistent and appropriate oral hygiene practices [23,24]. Varkey et al. [20] performed dental rehabilitation under general anesthesia on a 4-year-old child, meticulously planning extractions, several esthetic restorations, and space maintainers to avoid unnecessary bleeding. In this case, a seven-year-old child underwent extensive therapy under general anesthesia. Yadalam et al. [25] stated that obtaining a thorough medical history, taking precautions, and conducting a hematological pretreatment prior to referral to the dental office can successfully carry out periodontal treatment. However, in the current case, we only performed extractions and restorations as part of periodontal therapy. Rakocz et al. [26] performed the extraction of three teeth in three sessions in a 15-year-old girl with GT who had previously experienced severe bleeding and required platelet transfusions. The authors applied autologous fibrin glue locally to the extraction sites, along with tranexamic acid swish and swallow rinses. The authors did not use a systemic platelet infusion as a preventive measure. No postoperative bleeding occurred. Mehta and Bhatia [22] administered a dental treatment under local anesthesia to a 6-year-old girl with GT; they split the artificial teeth with AB gel. The authors opined that the pediatric dentist also plays an important role in advising and maintaining excellent oral hygiene and periodic oral assessment, thereby preventing any dental health problems and providing an improved quality of life for the patient [22,26,27]. However, in this case, the treatment took place under general anesthesia without the use of a splint. The role of the pediatric dentist lies in giving proper anticipatory guidance to parents of children with GT with regard to avoiding traumatic injuries, providing proper periodontal care, and encouraging optimal oral hygiene maintenance pertaining to both local and systemic health in such children.

## Conclusions

Patients with bleeding disorders may face complications when managing dental issues. However, careful coordination between pediatric dentists and hematologists can achieve satisfactory hemostasis, minimizing platelet concentrations. Preoperative hematological evaluations and platelet transfusions are not necessary and essential in all cases. Postoperatively, daily assessments of Hb levels and oral hygiene maintenance should be performed. An appropriate treatment plan with the utmost care, especially under GA, will be beneficial to patients with GT.
